# Practical Points

**Published:** 1894-11-03

**Authors:** 


### PRACTICAL POINTS.

*Temporary Hospitals and their Construction.—A correspondent writes to inquire the best place to obtain an iron, wood-lined surgical room and fittings to attach to a cottage hospital. Who would be the best people to apply to with reasonable security of adequate lighting and heating being provided? Is there any firm which makes a speciality of such temporary hospitals?*

We assume that surgical-room means an operation-room. Probably Humphrey and Co. (Limited), of Knightsbridge, are the best makers of iron houses; but we think for an operation-room the Wire-wove, Waterproof Roofing Company, 108, Queen Victoria Street, would be the best firm to employ. The temporary operation-room at the Royal Infirmary, Derby, has been carried out in this material, and is most satisfactory.

